# Supplementary material for: Comparative Investigation of Raw and Processed Radix Polygoni Multiflori on the Treatment of Vascular Dementia by Liquid Chromatograph−Mass Spectrometry Based Metabolomic Approach
Source: Metabolites. 2022 Dec 19;12(12):1297. doi: 10.3390/metabo12121297 (PMC9785642; doi:10.3390/metabo12121297)
Supplement: Supplementary file 1 [file metabolites-12-01297-s001.zip › metabolites-2069680-supplementary.pdf]

metabolites

MDPI

Supplementary Materials

Analysis of PPM and RPM extract components

The samples of PPM and RPM extract were prepared by adding methanol at a concentration of 1 mg/ml. The samples were centrifuged at 12000 rpm for 15 min at 4°C. The supernatant was analyzed by LC−MS.

For LC−MS analysis, ultrahigh-performance liquid chromatography–qudrupole time-of-flight mass spectrometry (AB SCIEX Triple TOF 5600+, USA) equipped. The separation was performed by gradient elution using mobile phase A (0.1% formic acid) and mobile phase B (100% methanol). Separation was achieved on a Waters Atlantis™ Premier BEH C18 AX column (100 × 2.1 mm, 1.7 μm) at a flow rate of 0.3 mL/min with a gradient elution using 0.1% formic acid in water as mobile phase A and acetonitrile as mobile phase B. The flow rate was 0.3 mL/min, with an autosampler temperature of 4°C and an injection volume of 2 μL.

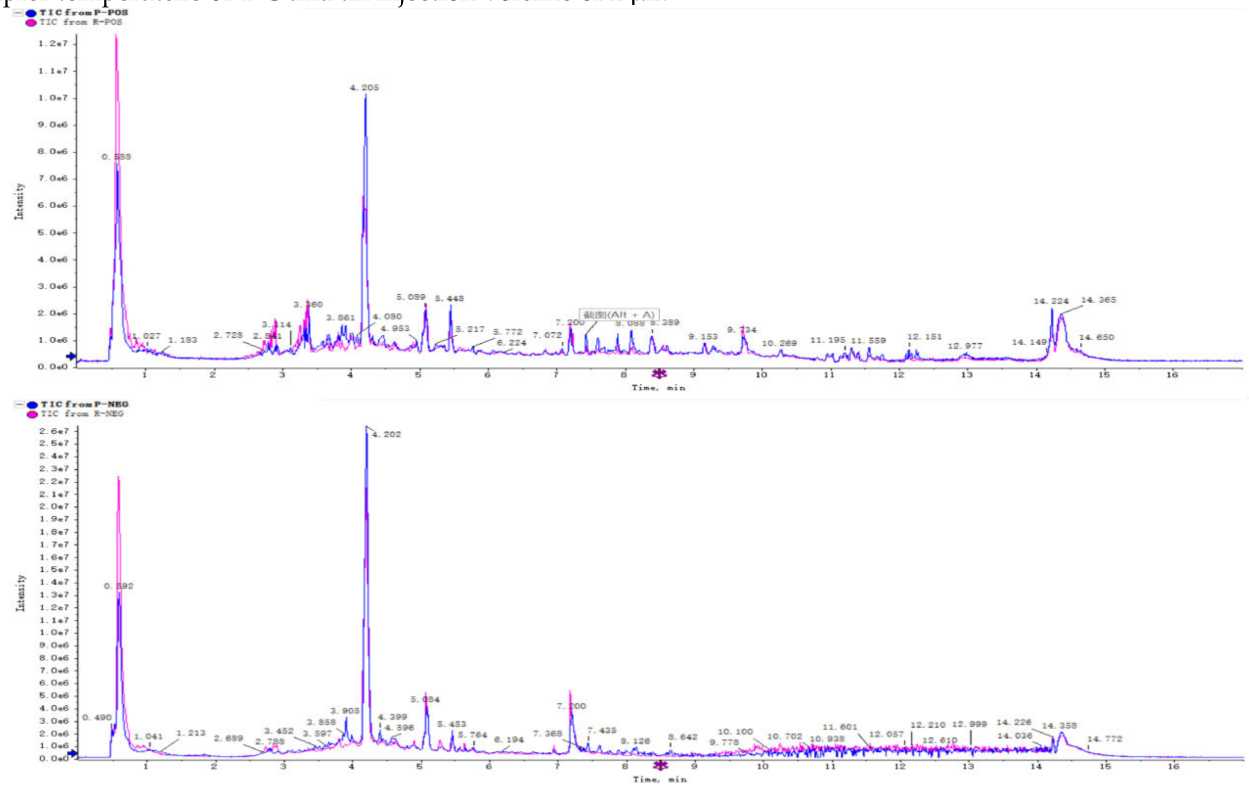

Figure S1. Total ion chromatogram of extract of PPM and RPM

By comparison with the database, a total of 29 compounds were identified in the extract of PPM, and 25 compounds were identified in the extract of RPM, as shown in Table S1, 2.

Table S1. Compounds assigned in extract of PPM.

Component Name	Area	RT (min)	Adduct / Charge	Formula	Precursor Mass	Mass Error (ppm)
Icaritin	4.82E+04	0.56	[M - H]-	C21H20O6	367.1214	3.5
5,6,7,8-Tetrahydroxy-2-(4-hydroxy-3,5-dimethoxyphenyl)chromen-4-one	4.81E+05	0.57	[M + H2O + H]+	C17H14O9	381.0793	-4.4

Butanedioic Acid	5.97E+03	0.60	[M + H3N + H]+	C4H6O4	136.0617	-3.2
(-)-Catechin	8.83E+04	3.45	[M - H]-	C15H14O6	289.0720	0.8
Mulberroside A	1.61E+04	3.59	[M - H2O + H]+	C26H32O14	551.1749	-2
2,5-Bis(Beta-D-Glucopyranosyloxy)-3,4'-Dihydroxystilbene	1.20E+05	3.64	[M - H]-	C26H32O14	567.1723	2.6
3-(Hydroxymethyl)tyrosine	1.25E+04	3.70	[M - H]-	C10H13NO4	210.0769	-1.8
D-Glucose, 2-O-[2,4-dihydroxy-6-[2-(4-hydroxyphenyl)ethenyl] phenyl]-	2.31E+06	3.90	[M - H]-	C20H22O9	405.1187	-0.9
3-acetyl-7-methoxy-2-methyl-5-[(2S,3R,4S,5S,6R)-3,4,5-trihydroxy-6-(hydroxymethyl)oxan-2-yl]oxynaphthalene-1,4-dione	1.02E+05	3.94	[M-H]-	C20H22O10	421.1145	1.1
(2S,3R,4R,5S,6R)-2-[5-hydroxy-3-[(E)-2-(4-hydroxyphenyl)ethenyl]-2-[(2S,3R,4R,5S,6R)-3,4,5-trihydroxy-6-(hydroxymethyl)oxan-2-yl]oxyphenoxy]-6-(hydroxymethyl)oxane-3,4,5-triol	7.45E+03	3.99	[M - H]-	C26H32O14	567.1720	0.2
3-Pyrrolidineacetic acid, 2-carboxy-4-(1-methylethenyl)-	1.39E+04	4.03	[M - H]-	C10H15NO4	212.0932	0.8
5-(4-Hydroxyphenyl)peroxybenzene-1,3-diol	2.06E+04	4.13	[M - H]-	C12H10O5	233.0460	1.5
2-Methoxystypandrone	6.18E+03	4.19	[M - H]-	C14H12O5	259.0621	3.5
2,3,5,4'-Tetrahydroxystilbene 2-O-Beta-D-Glucoside	1.69E+06	4.20	[M + H]+	C20H22O9	407.1334	-0.9
2,3,5,4''-Tetrahydroxystilbene 2-O-glucopyranoside	1.17E+07	4.20	[M-H]-	C20H22O9	405.1190	-0.3
Astringenin;trans-Piceatannol	3.40E+04	4.20	[M - H]-	C14H12O4	243.0667	1.3
Flavosativaside	2.44E+04	4.67	[M - H]-	C27H30O15	593.1503	-1.4
Resveravine(R)	1.21E+04	4.85	[M - H]-	C14H12O3	227.0724	4.1
7-hydroxy-4,5-dimethylchromen-2-one	9.51E+04	4.87	[M - H]-	C11H10O3	189.0560	-0.2
Moupinamide	2.28E+04	4.96	[M - H]-	C18H19NO4	312.1246	1.4
Torachrysone 8-O-Glucoside	1.24E+05	5.04	[M - H]-	C20H24O9	407.1360	3
Emodin-8-glucoside	1.11E+06	5.06	[M - H]-	C21H20O10	431.0990	1.5
9,10-Anthracenedione, 1,2,3-trihydroxy-8-methoxy-6-methyl-	4.73E+03	5.46	[M - H]-	C16H12O6	299.0575	4.3
Physcion 8-beta-D-glucoside	1.20E+05	5.46	[M - H]-	C22H22O10	445.1141	0.3
5-Heptenoic acid, 7-[(1S,3R,4S,5S)-3-[(1E,3S)-3-hydroxy-1-octen-1-yl]-2,6-dioxabicyclo[3.1.1]hept-4-yl]-, (5Z)-	1.59E+04	5.72	[M + H]+	C20H32O5	353.2312	-3
Methyl 4,5-didehydrojasmonate	1.03E+04	6.10	[M - H]-	C13H18O3	221.1185	0.9
emodin	1.75E+05	7.19	[M + H]+	C15H10O5	271.0600	-0.7
1,6-dihydroxy-8-methoxy-3-methylanthracene-9,10-dione	1.98E+04	8.39	[M - H]-	C16H12O5	283.0625	0.3
9,12-Octadecadienoic Acid	9.45E+04	9.77	[M - H]-	C18H32O2	279.2335	2

Table S2. Compounds assigned in extract of RPM.

Component Name	Area	RT (min)	Adduct / Charge	Formula	Precursor Mass	Mass Error (ppm)
5,6,7,8-Tetrahydroxy-2-(4-hydroxy-3,5-dimethoxyphenyl)chromen-4-one	1.72E+06	0.57	[M + H2O + H]+	C17H14O9	381.0793	-4.4
Butanedioic Acid	6.98E+03	0.60	[M + H3N + H]+	C4H6O4	136.0617	-3.2
(-)-Catechin	7.01E+04	3.45	[M - H]-	C15H14O6	289.0720	0.8

Mulberroside A	1.26E+04	3.59	[M - H2O + H]+	C26H32O14	551.1749	-2
2,5-Bis(Beta-D-Glucopyranosyloxy)-3,4'-Dihydroxystilbene	1.82E+03	3.64	[M - H]-	C26H32O14	567.1723	0.4
3-(Hydroxymethyl)tyrosine	4.31E+01	3.70	[M - H]-	C10H13NO4	210.0769	-1.8
D-Glucose, 2-O-[2,4-dihydroxy-6-[2-(4-hydroxyphenyl)ethenyl] phenyl]-	1.35E+03	3.90	[M - H]-	C20H22O9	405.1187	-0.9
3-acetyl-7-methoxy-2-methyl-5-[(2S,3R,4S,5S,6R)-3,4,5-trihydroxy-6-(hydroxymethyl)oxan-2-yl]oxynaphthalene-1,4-dione	1.42E+04	3.94	[M-H]-	C20H22O10	421.1145	1.1
3-Pyrrolidineacetic acid, 2-carboxy-4-(1-methylethenyl)-	3.92E+03	4.03	[M - H]-	C10H15NO4	212.0932	0.8
5-(4-Hydroxyphenyl)peroxybenzene-1,3-diol	4.89E+03	4.13	[M - H]-	C12H10O5	233.0460	1.5
2-Methoxystypandrone	7.87E+03	4.19	[M - H]-	C14H12O5	259.0621	3.5
2,3,5,4'-Tetrahydroxystilbene 2-O-Beta-D-Glucoside	1.69E+06	4.20	[M + H]+	C20H22O9	407.1334	-0.9
2,3,5,4''-Tetrahydroxystilbene 2-O-glucopyranoside	8.04E+06	4.20	[M-H]-	C20H22O9	405.1190	-0.3
Astringenin;trans-Piceatannol	2.32E+04	4.20	[M - H]-	C14H12O4	243.0667	1.3
Flavosativaside	1.73E+04	4.67	[M - H]-	C27H30O15	593.1503	-1.4
7-hydroxy-4,5-dimethylchromen-2-one	9.08E+03	4.87	[M - H]-	C11H10O3	189.0560	-0.2
Moupinamide	1.94E+04	4.96	[M - H]-	C18H19NO4	312.1246	1.4
Torachrysone 8-O-Glucoside	1.56E+05	5.04	[M - H]-	C20H24O9	407.1360	3
Emodin-8-glucoside	2.19E+03	5.06	[M - H]-	C21H20O10	431.0990	1.5
9,10-Anthracenedione, 1,2,3-trihydroxy-8-methoxy-6-methyl-	3.54E+03	5.46	[M - H]-	C16H12O6	299.0575	4.3
5-Heptenoic acid, 7-[(1S,3R,4S,5S)-3-[(1E,3S)-3-hydroxy-1-octen-1-yl]-2,6-dioxabicyclo[3.1.1]hept-4-yl]-, (5Z)-	3.01E+04	5.72	[M + H]+	C20H32O5	353.2312	-3
Methyl 4,5-didehydrojasmonate	1.16E+04	6.10	[M - H]-	C13H18O3	221.1185	0.9
emodin	2.39E+05	7.19	[M + H]+	C15H10O5	271.0600	-0.7
1,6-dihydroxy-8-methoxy-3-methylanthracene-9,10-dione	1.08E+04	8.39	[M - H]-	C16H12O5	283.0625	0.3
9,12-Octadecadienoic Acid	2.39E+05	9.77	[M - H]-	C18H32O2	279.2335	2
